# Stevens-Johnson Syndrome as a Risk Factor for Ocular Surface Squamous Neoplasia in a Pediatric Patient

**DOI:** 10.7759/cureus.83919

**Published:** 2025-05-11

**Authors:** Mauricio Muleiro-Alvarez, Angelica Hernandez-Solis, Gustavo Ortiz-Morales, Alejandro Navas, Nicolás Kahuam-López, Arturo Ramirez-Miranda, Enrique O. Graue-Hernandez

**Affiliations:** 1 Cornea and Refractive Surgery, Instituto de Oftalmologia Fundacion Conde de Valenciana-The International Agency for the Prevention of Blindness (IAP), Mexico City, MEX

**Keywords:** limbal stem cell deficiency, ocular surface squamous neoplasia, simple limbal epithelial transplant, slet, stevens-johnson syndrome

## Abstract

This report describes a rare case of ocular surface squamous neoplasia (OSSN) developing in the aftermath of Stevens-Johnson syndrome (SJS). A 15-year-old female patient, who had previously experienced an episode of SJS triggered by non-steroidal anti-inflammatory drugs (NSAIDs), developed limbal stem cell deficiency (LSCD) and was subsequently diagnosed with OSSN in the setting of chronic ocular surface inflammation. She was treated successfully with topical immunotherapy using interferon alpha-2b. This case highlights the pivotal role of chronic inflammation and immune dysregulation in driving neoplastic transformation of the ocular surface. In particular, severe immune-mediated disorders such as SJS can disrupt corneal homeostasis and establish a pro-oncogenic microenvironment that fosters dysplasia and neoplastic progression.

## Introduction

Ocular surface squamous neoplasia (OSSN) encompasses a spectrum of pathologies characterized by dysplastic squamous epithelial cells in the conjunctiva, cornea, or limbus [1]. Although it is the most common cause of non-pigmented tumors on the ocular surface, its incidence remains low, ranging from 0.03 to 3.4 cases per 100,000 individuals annually [2]. However, its occurrence in pediatric patients is exceedingly rare, with limited documentation in the literature.

Several well-established risk factors contribute to the development of OSSN, including exposure to petroleum products, fair skin and hair, smoking, and xerophthalmia [3]. However, the primary risk factors are human immunodeficiency virus (HIV), ultraviolet-B (UVB) radiation, and human papillomavirus (HPV) [3]. Chronic ocular surface inflammation is also a significant predisposing factor for metaplastic changes, potentially leading to dysplasia and neoplasia [4].

We present a case of a pediatric patient who developed OSSN following SJS, highlighting the interplay of chronic inflammation, limbal stem cell deficiency, and neoplastic transformation.

## Case presentation

A 15-year-old female patient presented to our Cornea Service with complaints of redness, photophobia, and visual disturbances in her left eye (OS). Her medical history included asthma and SJS at age four, triggered by non-steroidal anti-inflammatory drugs (NSAID) use. During the acute phase of SJS, she underwent amniotic membrane transplantation (AMT) in both eyes. Due to severe limbal stem cell deficiency (LSCD), an allogeneic simple limbal epithelial transplant (Allo-SLET) was performed on the OS at age 14, using donor tissue from her sibling. She has since been maintained on systemic immunosuppression (tacrolimus, prednisone, and mycophenolate mofetil).

**Figure 1:**
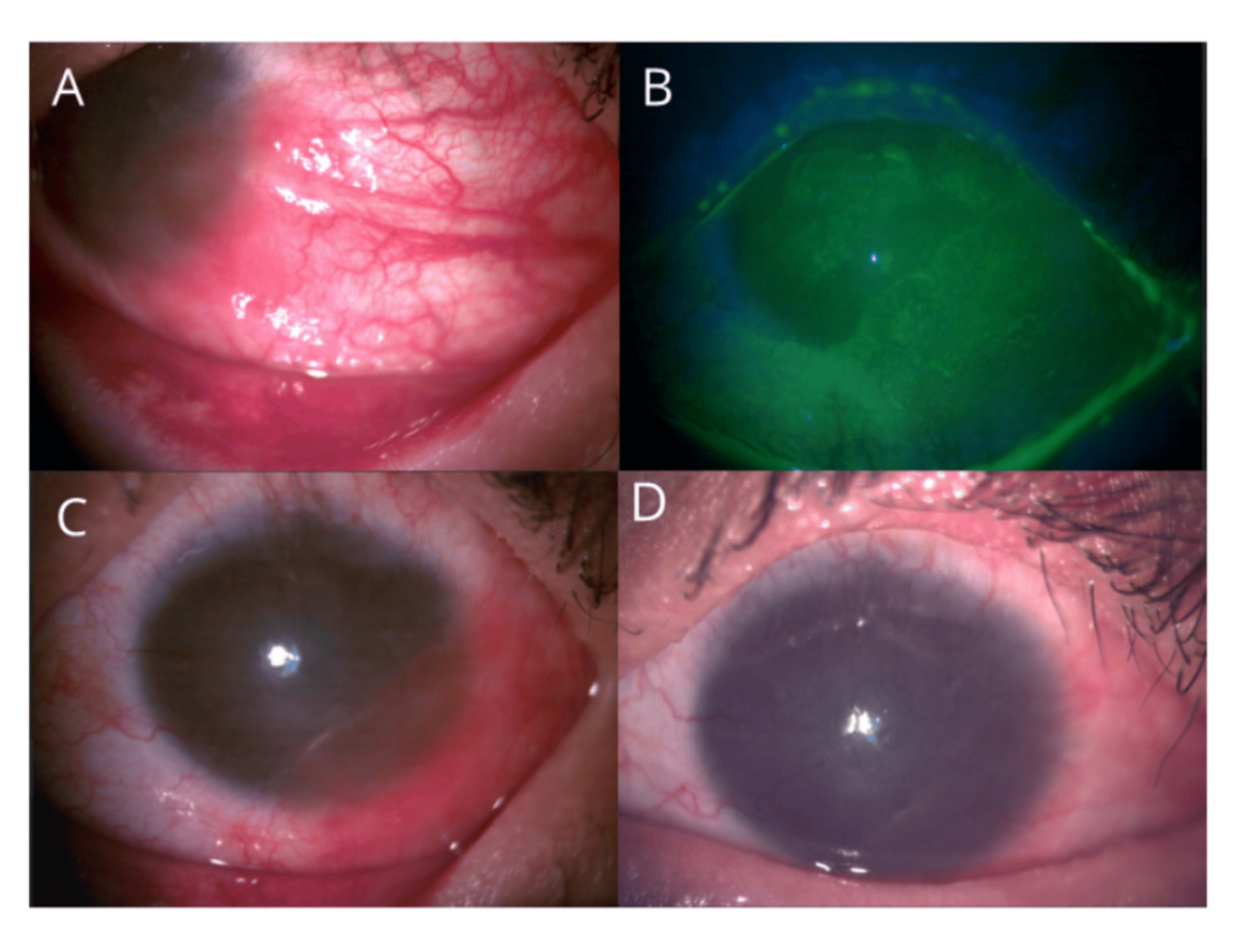
Slit-lamp photographs of the left eye showing the clinical course of the ocular surface squamous neoplasia (OSSN) (A) Initial presentation revealing a papillomatous, elevated, and vascularized lesion originating from the inferotemporal limbus and partially invading the cornea and visual axis. (B) Close-up view of the lesion demonstrating surface keratinization, irregular borders, and feeder vessels. (C) The alternative angle of the lesion further highlights its limbal origin and corneal extension before topical interferon α-2b therapy initiation. (D) Final appearance after five cycles of topical interferon α-2b, showing marked regression of the lesion, reduced vascularization, and restoration of corneal clarity.

On biomicroscopic examination, the right eye (OD) exhibited a conjunctival granuloma, diffuse pigmentation, hyperemic tarsal conjunctiva, superior conjunctivalization, and 360° LSCD. The OS revealed a hyperemic conjunctiva and an elevated ocular surface lesion originating from the lower temporal limbus, partially invading the visual axis and exhibiting fluorescein staining (Figure 1).

Uncorrected visual acuity (UCVA) was 20/400 in both eyes, with best-corrected visual acuity (BCVA) of 20/200 using a scleral contact lens. The patient was prescribed preservative-free sodium hyaluronate 0.15% hourly, dexpanthenol 5% every eight hours, fluorometholone 0.1% every six hours (tapered weekly), and topical interferon α-2b every six hours for one month, completing five cycles. At her final follow-up, significant clinical improvement was noted in the OS (Figure 1).

## Discussion

OSSN is rare in pediatric patients, yet its development in this case underscores the role of chronic inflammation in neoplastic progression. Persistent ocular inflammation disrupts homeostasis, facilitates cellular dysfunction, and fosters a microenvironment conducive to metaplasia and dysplasia [4].

OSSN is commonly observed in immunocompromised individuals, such as those with HIV or xeroderma pigmentosum [5]. However, in this case, the patient’s chronic immune-mediated conditions, SJS and LSCD, likely contributed to OSSN development. LSCD, a frequent complication of SJS, leads to conjunctivalization, corneal opacity, and progressive vision loss. In severe cases, corneal homeostasis can be restored through limbal stem cell transplantation, either autologous SLET (Auto-SLET) or Allo-SLET. While Allo-SLET is effective, it increases the risk of immune rejection and necessitates long-term immunosuppression, which can further disrupt immune surveillance and promote neoplastic changes [6-8].

Another potential etiology, although unlikely given the donor’s age, for OSSN in this patient is the theoretical transmission of neoplastic cells through Allo-SLET. While tumor transmission has been documented in keratolimbal allografts, no such cases have been reported following SLET [9,10]. 

In managing OSSN, medical therapy was prioritized to minimize surgical trauma and preserve ocular surface integrity. Interferon α-2b has been shown to induce OSSN regression without necessitating surgical excision, which carries the risk of additional ocular surface damage [1]. Following five cycles of treatment, significant lesion regression was achieved.

This case underscores the importance of long-term monitoring in patients with SJS, as chronic inflammation and limbal dysfunction increase their susceptibility to severe ocular complications, including OSSN. It also highlights the need for an integrated management approach that balances immunosuppressive therapy with vigilant neoplastic surveillance.

This case illustrates the complex interplay between chronic inflammation, LSCD, and OSSN. In particular, it underscores the necessity of long-term surveillance in SJS patients, who may be at heightened risk for neoplastic transformation. The successful resolution of OSSN with interferon α-2b further highlights the role of targeted immunotherapy in managing these challenging cases.

## Conclusions

OSSN is an exceptionally rare finding in pediatric patients, yet this case highlights its potential development in the setting of chronic immune-mediated ocular disease. The combination of SJS and LSCD likely disrupted ocular surface homeostasis, creating an environment conducive to neoplastic transformation. The effective resolution of OSSN using topical interferon α-2b underscores the value of non-invasive immunotherapy in preserving ocular integrity. This case reinforces the importance of long-term surveillance and multidisciplinary management in patients with chronic ocular surface inflammation, particularly those with a history of SJS. Further studies and case aggregation are warranted to better understand the incidence, risk factors, and optimal monitoring strategies for OSSN in pediatric populations with immune-mediated ocular surface disease.

## References

[1] Yeoh CH, Lee JJ, Lim BX (2022). The management of ocular surface squamous neoplasia (OSSN). Int J Mol Sci.

[2] Hollhumer R, Williams S, Michelow P (2020). Ocular surface squamous neoplasia: population demographics, pathogenesis and risk factors. Afr Vision Eye Health.

[3] Hӧllhumer R, Michelow P, Williams S (2023). Demographics, clinical presentation and risk factors of ocular surface squamous neoplasia at a tertiary hospital, South Africa. Eye (Lond).

[4] De Arrigunaga S, Wall S, Theotoka D (2024). Chronic inflammation as a proposed risk factor for ocular surface squamous neoplasia. Ocul Surf.

[5] Vempuluru VS, Ganguly A, Kaliki S (2021). Ocular surface squamous neoplasia following keratoplasty in xeroderma pigmentosa: a series of seven cases. Curr Eye Res.

[6] Liu Y, Feng J, Ren Y (2023). Ocular surface involvement and histopathologic changes in the acute stage of Stevens-Johnson syndrome and toxic epidermal necrolysis: a cross-sectional study. BMC Ophthalmol.

[7] Shanbhag SS, Patel CN, Goyal R, Donthineni PR, Singh V, Basu S (2019). Simple limbal epithelial transplantation (SLET): review of indications, surgical technique, mechanism, outcomes, limitations, and impact. Indian J Ophthalmol.

[8] Hama N, Aoki S, Chen CB (2024). Recent progress in Stevens-Johnson syndrome/toxic epidermal necrolysis: diagnostic criteria, pathogenesis and treatment. Br J Dermatol.

[9] Sepsakos L, Cheung AY, Nerad JA, Mogilishetty G, Holland EJ (2017). Donor-derived conjunctival-limbal melanoma after a keratolimbal allograft. Cornea.

[10] Miller AK, Young JW, Wilson DJ, Dunlap J, Chamberlain W (2017). Transmission of donor-derived breast carcinoma as a recurrent mass in a keratolimbal allograft. Cornea.

